# Enhanced Vitreous Imaging in Healthy Eyes Using Swept Source Optical Coherence Tomography

**DOI:** 10.1371/journal.pone.0102950

**Published:** 2014-07-18

**Authors:** Jonathan J. Liu, Andre J. Witkin, Mehreen Adhi, Ireneusz Grulkowski, Martin F. Kraus, Al-Hafeez Dhalla, Chen D. Lu, Joachim Hornegger, Jay S. Duker, James G. Fujimoto

**Affiliations:** 1 Massachusetts Institute of Technology, Department of Electrical Engineering and Computer Science, and Research Laboratory of Electronics, Cambridge, Massachusetts, United States of America; 2 New England Eye Center, Tufts Medical Center, Boston, Massachusetts, United States of America; 3 University of Erlangen-Nuremburg, Pattern Recognition Lab, and Graduate School in Advanced Optical Technologies (SAOT), Erlangen, Germany; Massachusetts Eye & Ear Infirmary, Harvard Medical School, United States of America

## Abstract

**Purpose:**

To describe enhanced vitreous imaging for visualization of anatomic features and microstructures within the posterior vitreous and vitreoretinal interface in healthy eyes using swept-source optical coherence tomography (SS-OCT). The study hypothesis was that long-wavelength, high-speed, volumetric SS-OCT with software registration motion correction and vitreous window display or high-dynamic-range (HDR) display improves detection sensitivity of posterior vitreous and vitreoretinal features compared to standard OCT logarithmic scale display.

**Design:**

Observational prospective cross-sectional study.

**Methods:**

Multiple wide-field three-dimensional SS-OCT scans (500×500A-scans over 12×12 mm^2^) were obtained using a prototype instrument in 22 eyes of 22 healthy volunteers. A registration motion-correction algorithm was applied to compensate motion and generate a single volumetric dataset. Each volumetric dataset was displayed in three forms: (1) standard logarithmic scale display, enhanced vitreous imaging using (2) vitreous window display and (3) HDR display. Each dataset was reviewed independently by three readers to identify features of the posterior vitreous and vitreoretinal interface. Detection sensitivities for these features were measured for each display method.

**Results:**

Features observed included the *bursa premacularis* (BPM), area of Martegiani, Cloquet's/BPM septum, Bergmeister papilla, posterior cortical vitreous (hyaloid) detachment, papillomacular hyaloid detachment, hyaloid attachment to retinal vessel(s), and granular opacities within vitreous cortex, Cloquet's canal, and BPM. The detection sensitivity for these features was 75.0% (95%CI: 67.8%–81.1%) using standard logarithmic scale display, 80.6% (95%CI: 73.8%–86.0%) using HDR display, and 91.9% (95%CI: 86.6%–95.2%) using vitreous window display.

**Conclusions:**

SS-OCT provides non-invasive, volumetric and measurable *in vivo* visualization of the anatomic microstructural features of the posterior vitreous and vitreoretinal interface. The vitreous window display provides the highest sensitivity for posterior vitreous and vitreoretinal interface analysis when compared to HDR and standard OCT logarithmic scale display. Enhanced vitreous imaging with SS-OCT may help assess the natural history and treatment response in vitreoretinal interface diseases.

## Introduction

The vitreous is a transparent hydrophilic gel, principally composed of water, occupying the space between the lens at the front of the eye and the retina lining the back of the eye. The vitreous functions as a pathway for nutrients utilized by the lens, ciliary body, and retina, and provides structural support to the globe. Aging of the vitreous is characterized by liquefaction and the formation of liquefied vitreous pockets in a process known as synchysis. When vitreous liquefaction and weakening of vitreoretinal adhesion occur concurrently, the vitreous collapses in a process called syneresis, which eventually leads to posterior vitreous detachment (PVD). [1] In vivo imaging of the vitreous can be performed using ophthalmoscopy, slit lamp biomicroscopy, scanning laser ophthalmoscopy, ultrasonography, and optical coherence tomography (OCT). [2] However, due to its transparency, it remains difficult to reliably image the vitreous except in advanced disease.

OCT is a non-invasive, *in vivo* optical imaging modality analogous to ultrasound, except imaging is performed using echoes of light measured by low coherence optical interferometry. [3] Time-domain OCT has been used in the past to examine the vitreous in a number of diseases of the vitreomacular interface. [4] The advent of spectral-domain OCT (SD-OCT) technology has allowed better visualization of the vitreoretinal interface and posterior vitreous cortex through improved axial resolution, imaging speed, and signal-to-noise ratio. Currently, SD-OCT is being widely used to diagnose and manage a variety of macular diseases, including vitreoretinal interface disease processes such as vitreomacular traction (VMT), epiretinal membrane (ERM), lamellar holes, pseudoholes, and full thickness macular holes (FTMH). [5], [6], [7], [8]

The development of swept-source OCT (SS-OCT) technology has enabled higher speeds, better sensitivity with imaging depth, and longer imaging range compared to SD-OCT. [9] Unlike SD-OCT, SS-OCT does not suffer from loss of sensitivity across the imaging range and the tissue of interest does not need to be positioned close to the zero delay to enhance sensitivity, as in enhanced depth imaging (EDI). [10] Recent SS-OCT studies have investigated the prevalence of the *bursa premacularis* (BPM) and area of Martegiani and measured the dimensions of the BPM in 2 dimensions using single B-scan imaging protocols. [11], [12] However, analysis of the posterior vitreous in 3 dimensions using volumetric imaging with high A-scan density has not yet been described using OCT.

OCT images are typically displayed in logarithmic scale in order to accommodate the large dynamic range of the backscattered light from the retina and vitreous. A typical dynamic range of an OCT image in the eye is ∼40 dB, while the detection sensitivity can be as high as ∼100 dB. [13] Since a limited number of grey scale levels can be displayed in an image and perceived by the human eye, logarithmic scale display is used to compress the dynamic range, resulting in loss of contrast within specific tissue structures. Similar to X-ray computed tomography (CT), where tissues of various densities such as bone, soft-tissue, liver, and lung can be viewed by changing contrast windows, OCT images can also be “windowed” to improve visualization of the vitreous structure. [11], [14], [15] Another complementary approach is tone mapping using adaptive histogram equalization algorithms to convert high-dynamic-range (HDR) images to a displayable range while preserving contrast, brightness, and fine detail. [16]

The present study describes imaging of the normal vitreous architecture with wide-field three-dimensional OCT (3D-OCT) using a prototype long-wavelength SS-OCT instrument, along with the use of both windowing and HDR techniques for an enhanced visualization of the anatomic and microstructural features of the vitreous. The aims of this pilot study were: (1) to describe long-wavelength, high-speed, volumetric SS-OCT methods for enhanced vitreous imaging, (2) to examine *in vivo* volumetric details of the vitreous structure in healthy eyes with wide-field 3D-OCT scans covering the fovea and optic nerve head regions, (3) to evaluate detection sensitivity of posterior vitreous and vitreoretinal features using windowing and HDR versus standard logarithmic scale OCT imaging.

## Materials and Methods

A research prototype high-speed SS-OCT instrument was designed and built that achieves 100,000 axial scans per second, which is ∼4× faster imaging speed than standard clinical SD-OCT instruments, with comparable <6 µm axial image resolution. This prototype SS-OCT instrument uses ∼1050 nm wavelength light which has better immunity to ocular opacity and improved image penetration into the choroid compared to standard clinical OCT instruments, which image with ∼840 nm light. [9], [17] This prototype SS-OCT has similar specifications to the recently introduced commercial instrument, the Topcon Deep Range Imaging (DRI) OCT-1 Atlantis 3D SS OCT (Topcon Medical Systems, Oakland, N.J.) which is not yet approved for use in the United States.

Healthy volunteers from the New England Eye Center at Tufts Medical Center (Boston, MA) were prospectively enrolled in the study, which was approved by the institutional review boards at Tufts Medical Center and Massachusetts Institute of Technology. The research adhered to the Declaration of Helsinki and the Health Insurance Portability and Accountability Act. Signed written informed consent was obtained from all participants.

Twenty-two eyes of 22 healthy subjects with normal vision and no history of retinal disease, optic nerve abnormalities, or ocular surgery were imaged at the New England Eye Center at Tufts Medical Center. One randomly-selected eye for each subject was imaged using the prototype long-wavelength, high-speed SS-OCT instrument. Up to eight orthogonally raster scanned 3D-OCT datasets were acquired in each eye over a 12 mm×12 mm retinal area (∼40°) with 500 A-scans × 500 B-scans centered between the fovea and the optic nerve by a single trained operator. The acquisition time per 3D-OCT dataset was less than 3 seconds. A registration motion-correction algorithm was applied to remove motion artifacts in the volumetric data. A minimum of two orthogonally scanned 3D-OCT volumetric datasets were merged to create a single, motion-corrected volumetric dataset with improved signal. Arbitrary OCT images from the 3D-OCT volumetric dataset can be viewed in any two-dimensional plane. Details of the registration motion-correction algorithm have been described previously. [18]

**Figure 1:**
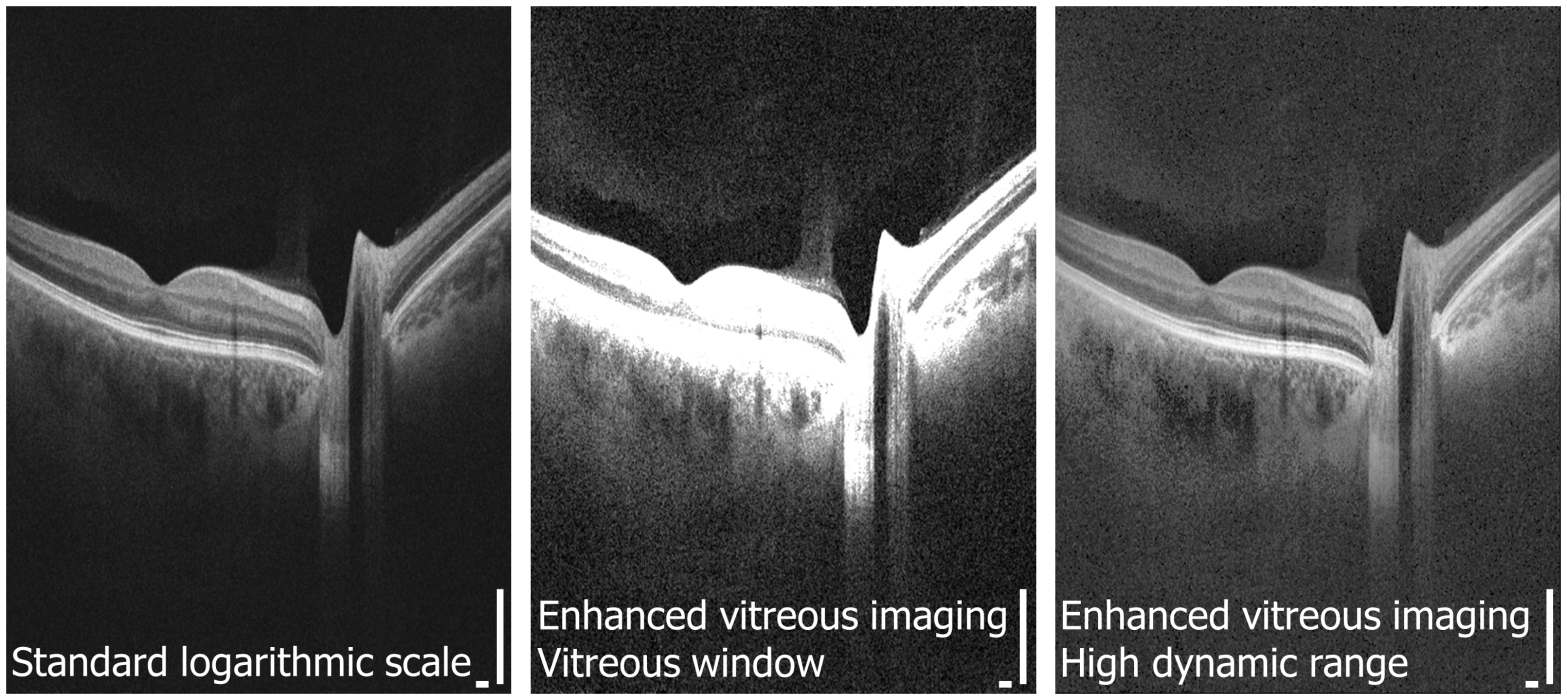
Optical coherence tomography (OCT) images are typically displayed in logarithmic scale. Enhanced vitreous imaging with the vitreous window and high-dynamic-range methods improves visualization of structure in the posterior vitreous and vitreoretinal interface. Scale bars: 300 µm.

**Figure 2:**
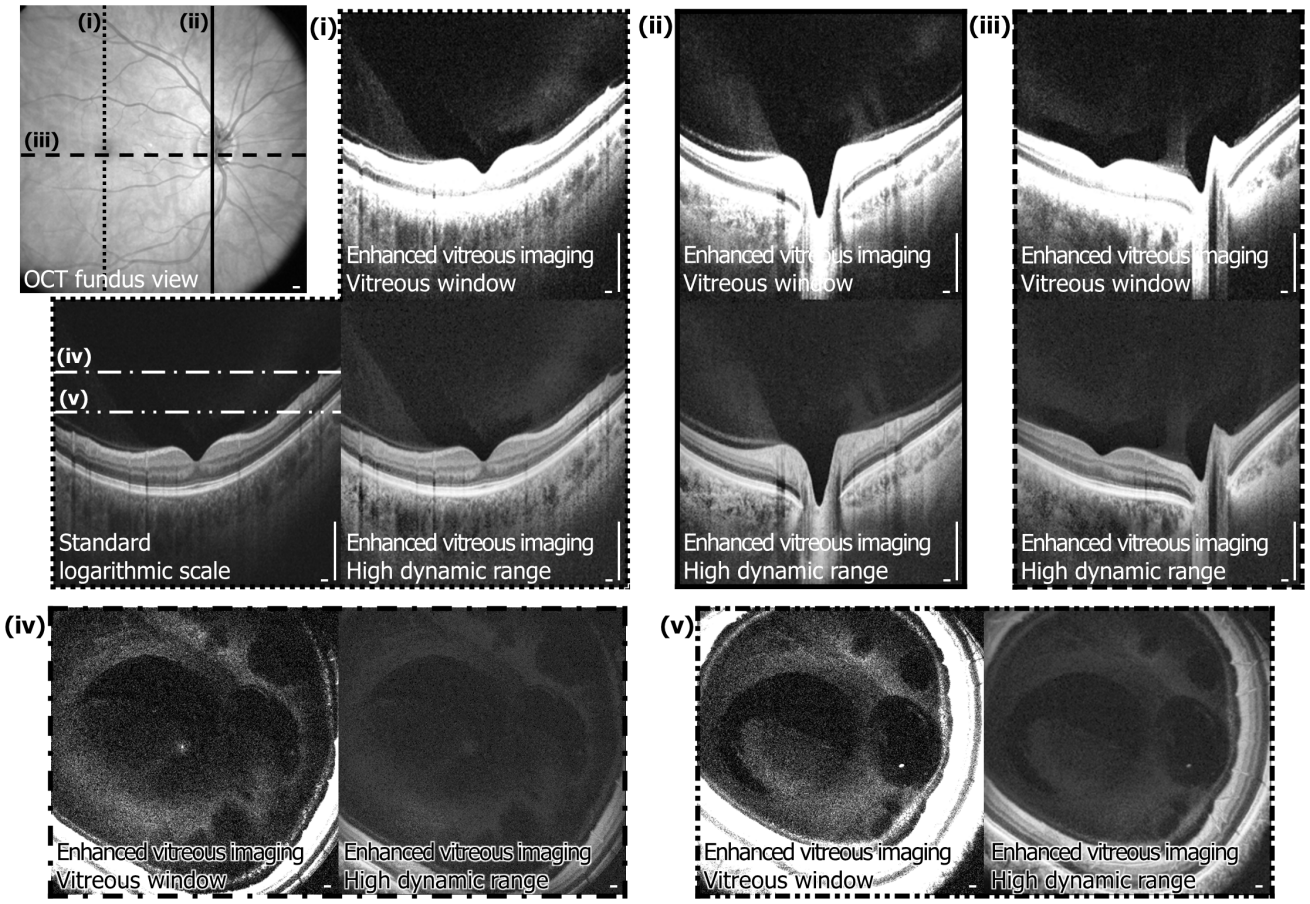
Three-dimensional (3D) enhanced vitreous imaging enables visualization of the posterior vitreous and vitreoretinal interface in arbitrary cross sections or any *en face* plane. Arbitrary cross-sectional images and arbitrary *en face* images are generated from the 3D motion-corrected volumetric dataset with high-speed SS-OCT enhanced vitreous imaging display. Videos S1 and S2 are cross-sectional and *en face* flythrough videos of the 3D volumetric dataset. Scale bars: 300 µm.

To enhance visualization of the posterior vitreous and vitreoretinal interface, each motion-corrected volumetric dataset was displayed using (1) the standard OCT logarithmic scale, (2) enhanced vitreous imaging using vitreous window display – a windowing method analogous to CT that was performed by adjusting the threshold and contrast of logarithmic scale OCT data in each motion-corrected volumetric dataset, [14] and (3) enhanced vitreous imaging using HDR display – an HDR filtering method that was performed by applying a contrast-limited adaptive histogram equalization (CLAHE) filter to the motion-corrected volumetric dataset in linear scale. [16] All enhanced vitreous imaging image processing was performed using ImageJ (NIH, Bethesda, MD) and its plugins. [19], [20] Figure 1 is an example of a motion-corrected dataset shown in the standard logarithmic scale display, the enhanced vitreous imaging vitreous window display, and the HDR display. Figure 2 demonstrates 3D OCT, where arbitrary cross sections as well as *en face* images extracted from the 3D volumetric dataset are shown. The hypothesis of this study was that SS-OCT enhanced vitreous imaging using the windowing or the HDR method improves reader detection sensitivity of vitreal and vitreoretinal features compared to standard OCT logarithmic scale display.

**Figure 3:**
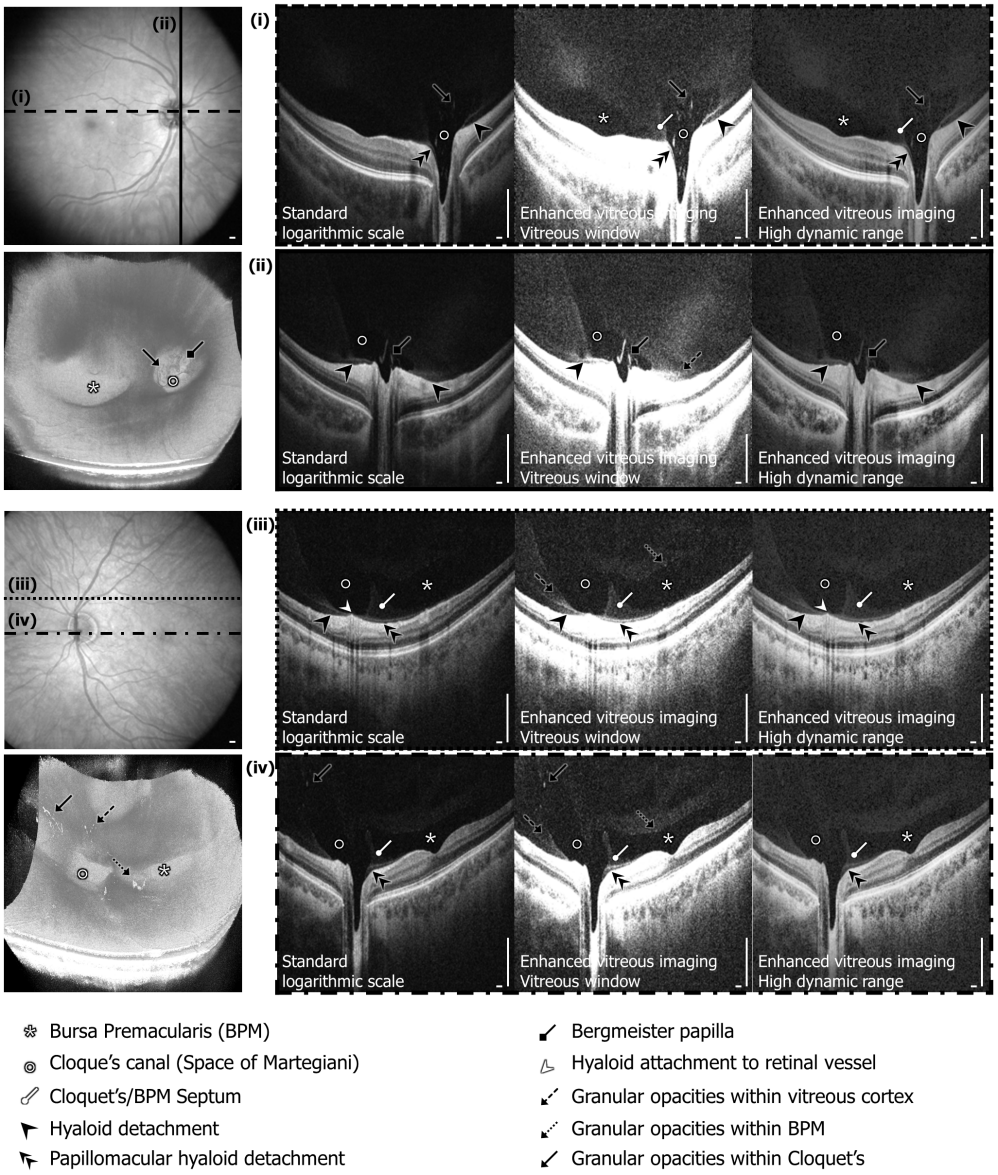
Examples of features observed in the posterior vitreous and vitreoretinal interface in healthy eyes. Selected cross sections from two different eyes are shown with their locations marked on the optical coherence tomography (OCT) fundus images. Renderings of the 3D volumetric datasets are also shown. Note the cloudy gray appearance of reflective signal from the vitreous, where liquefied areas of the vitreous appear transparent and hyperreflective foci appear white. Observed features are marked in the cross-sectional images: *bursa premacularis* (BPM) (white asterisk), Cloquet's canal (Area of Martegiani) (white circle), Cloquet's/BPM septum (white circle arrow), posterior cortical vitreous (hyaloid) detachment (black arrowhead), papillomacular hyaloid detachment (double black arrowheads), Bergmeister papilla (black diamond arrow), hyaloid attachment to retinal vessel (white arrowhead), granular opacities within vitreous cortex (black dashed arrow), granular opacities within BPM (black dotted arrow), granular opacities within Cloquet's (black arrow). Scale bars: 300 µm.

Each dataset, displayed in 3 forms (Figure 1), was reviewed independently by three expert OCT readers (J.J.L, A.J.W, and M.A. each with 1 year or more full-time ophthalmic OCT research experience in a PhD or research fellowship program). Datasets were reviewed in random order and separated by display methods, so that each dataset was examined independently for each display method. The presence of specific anatomic and microstructural features within the posterior vitreous and vitreoretinal interface was identified by examining multiple nasal-temporal cross-sectional images extracted from the 3D volumetric dataset. The readers first examined a subset of coarsely sampled 18 cross-sectional images over the entire field of view along with a subset of densely sampled 18 cross-sectional images over the macular and optic nerve head regions, followed by finer spaced sequential cross-sectional images if needed. Features studied included: the BPM, Cloquet's canal (area of Martegiani), Cloquet's/BPM septum, Bergmeister papilla, posterior cortical vitreous (hyaloid) detachment, papillomacular hyaloid detachment, hyaloid attachment to retinal vessel(s), granular opacities within vitreous cortex, granular opacities within Cloquet's canal, and granular opacities within the BPM. (Figure 3) Specific locations of microstructural features in each cross-sectional image were recorded by each reader. Disagreement between readers was resolved by open adjudication. The decision of whether to classify each feature as present or absent was determined by combining positive findings from the standard logarithmic scale display, enhanced vitreous imaging using vitreous window display, and HDR display in order to generate the maximum possible detection sensitivity for each feature. More specifically, since there does not exist a standard imaging method to detect all of the features investigated in this study, the true positive control was obtained by combined all reading results: if there was a positive result in any of the three display methods, the existence of the feature was considered true positive. The detection sensitivity of each display method for each vitreal and vitreomacular feature was calculated as the proportion of true positives correctly identified as such (Sensitivity  =  True Positives/(True Positives + False Negatives)). The 95% confidence interval (CI) for detection of all the vitreal and vitreoretinal features for each display method was also calculated. All statistical analysis was conducted using Excel (Microsoft Corporation, Redmond, WA).

To demonstrate the ability to analyze posterior vitreous structures in 3D, a motion-corrected volumetric dataset of a selected eye was processed using the vitreous window display. In the 3D dataset, the volumes of the BPM and area of Martegiani were measured, both which are optically transparent spaces within the vitreous. The same eye was used to demonstrate mapping of vitreous detachment from the retinal surface. Manual segmentation of the BPM and area of Martegiani and the manual mapping of vitreous detachment were performed using Amira (Visualization Sciences Group, Burlington, MA).

## Results

The mean age of the 22 healthy subjects was 33.0 years (range 23 to 49 years). There were 12 females (54.5%) and 10 males (45.5%). The features of the posterior vitreous and vitreoretinal interface observed with all display methods are shown in Table 1.

**Table 1:** Features of the posterior vitreous and vitreoretinal interface observed in healthy eyes.

Vitreal or Vitreoretinal Feature	Healthy Eyes with Feature n, (%)
Bursa Premacularis (BPM)	18 (81.8%)
Cloquet's canal (Area of Martegiani)	15 (68.2%)
Cloquet's/BPM Septum	17 (77.3%)
Bergmeister papilla	11 (50.0%)
Posterior cortical vitreous (hyaloid) detachment	20 (90.9%)
Papillomacular hyaloid detachment	16 (72.7%)
Hyaloid attachment to retinal vessel(s)	16 (72.7%)
Granular opacities within vitreous cortex	13 (59.1%)
Granular opacities within Cloquet's	17 (77.3%)
Granular opacities within BPM	17 (77.3%)

To test the hypothesis that SS-OCT enhanced vitreous imaging methods improve visualization of vitreal and vitreoretinal features, we compared standard logarithmic scale display OCT images to the two enhanced vitreous imaging methods. The sensitivity for detection of all vitreal and vitreoretinal features in this study was 75.0% (95% CI: 67.8%–81.1%) for the standard logarithmic scale display, 80.6% (95% CI: 73.8%–86.0%) for the HDR display and 91.9% (95% CI: 86.6%–95.2%) for the vitreous window display. The vitreous window display was able to detect an additional 34 (21.3%) features that were not visible on the standard logarithmic scale display and 30 (18.8%) features that were not visible on the HDR display. The HDR display detected an additional 25 (15.6%) features that were not visible on the standard logarithmic scale display and 10 (6.3%) features that were not visible on the vitreous window display. Three features (1.9%) were visible on the standard logarithmic scale display but not evident on either the vitreous window display or the HDR display.

The detection sensitivity for each of the vitreal and vitreoretinal features using the 3 display methods are shown in Table 2. The vitreous window display was more sensitive than the standard logarithmic scale display in detecting all the features. The HDR display was more sensitive than the standard logarithmic scale display in detecting the BPM (94.4% vs 77.8%), Cloquet's/BPM septum (100% vs 82.4%), Bergmeister papilla (81.8% vs 63.6%), posterior cortical vitreous (hyaloid) detachment (100% vs 90.0%), and hyaloid attachment to retinal vessel(s) (81.3% vs 68.8%). HDR had a comparable sensitivity to the standard logarithmic scale display in detecting area of Martegiani (93.3%), papillomacular hyaloid detachment (81.3%), granular opacities within Cloquet's (70.6%), and granular opacities within BPM (58.8%) and was less sensitive than the standard logarithmic scale display in detecting granular opacities within vitreous cortex (30.7% vs 53.8%). The vitreous window display had a higher or comparable sensitivity to the HDR display for detecting most vitreal and vitreoretinal features, except for detecting hyaloid attachment to retinal vessels and BPM, where the HDR display was more sensitive than the vitreous window display (81.3% vs 75.0% and 94.4% vs. 88.9% respectively).

**Table 2:** Detection of vitreal and vitreoretinal features in healthy eyes.

Observed Features	Sensitivity %, (95% Confidence Interval)		
	Standard OCT Logarithmic Scale Display	Vitreous Window Display	HDR Display
*Bursa Premacularis* (BPM)	77.8 (54.8 to 91.0)	88.9 (67.2 to 96.9)	94.4 (74.2 to 99.0)
Cloquet's canal (Area of Martegiani)	93.3 (70.2 to 98.8)	100.0 (79.6 to 100.0)	93.3 (70.2 to 98.8)
Cloquet's/BPM Septum	82.4 (59.0 to 93.8)	100.0 (81.6 to 100.0)	100.0 (81.6 to 100.0)
Bergmeister papilla	63.6 (35.4 to 84.8)	90.1 (62.3 to 98.4)	81.8 (52.3 to 94.9)
Posterior cortical vitreous (hyaloid) detachment	90.0 (69.9 to 97.2)	100.0 (83.9 to 100.0)	100.0 (83.9 to 100.0)
Papillomacular hyaloid detachment	81.3 (57.0 to 93.4)	93.8 (71.7 to 98.9)	81.3 (57.0 to 93.4)
Hyaloid attachment to retinal vessel(s)	68.8 (44.4 to 85.8)	75.0 (50.5 to 89.8)	81.3 (57.0 to 93.4)
Granular opacities within vitreous cortex	53.8 (29.1 to 76.8)	92.3 (66.7 to 98.6)	30.7 (12.7 to 57.6)
Granular opacities within Cloquet's	70.6 (46.9 to 86.7)	88.2 (65.7 to 96.7)	70.6 (46.9 to 86.7)
Granular opacities within BPM	58.8 (36.0 to 78.4)	88.2 (65.7 to 96.7)	58.8 (36.0 to 78.4)

OCT  =  Optical Coherence Tomography; HDR  =  High-dynamic-range.

**Figure 4:**
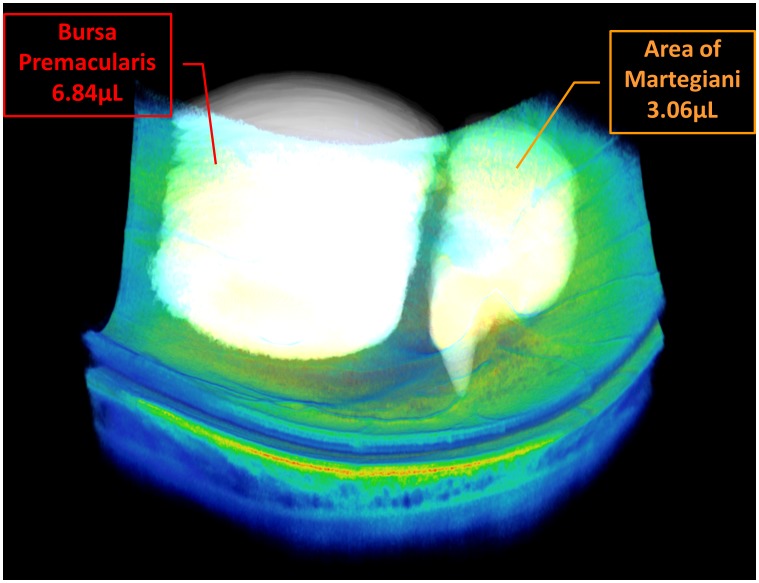
Volumetric measurement of vitreal spaces can be performed in three-dimensional (3D) enhanced vitreous imaging volumetric datasets. The *bursa premacularis* (BPM) and area of Martegiani are segmented and highlighted. The volume of the BPM and area of Martegiani measured within the imaging range of the dataset is 6.84 µL and 3.06 µL, respectively. Video S3 is a 3D rendering video animation of the retina with the highlighted BPM and area of Martegiani.

**Figure 5:**
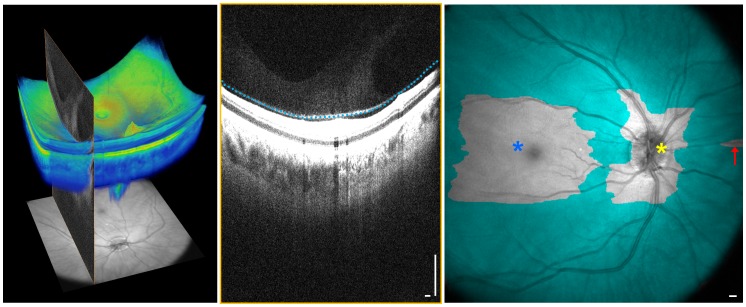
Vitreal detachment from the retina can be mapped in three-dimensional (3D) enhanced vitreous imaging volumetric datasets by examining each cross-sectional image in a 3D dataset (left) and marking the detached hyaloid (center) to generate a map where the area of vitreal detachment is highlighted (right). Vitreoretinal attachment is present at the macula (blue asterisk) and optic nerve head (yellow asterisk) as well as along a retinal vessel (red arrow) nasal to the optic nerve head. The measured areas of attachment are 20.9 mm^2^ above the macula, 9.7 mm^2^ over the optic nerve head, and 0.2 mm^2^ along the retinal vessel within the imaging range. Scale bars: 300 µm.

Using 3D information from a motion-corrected dataset of a selected eye processed using the vitreous window display, the BPM was measured to be 6.84 µL and the area of Martegiani was measured to be 3.06 µL (Figure 4). A vitreous detachment map was also generated for this eye, which illustrates remaining vitreal attachment over the macula and optic nerve head region as well as over a retinal vessel nasal to the optic nerve head (Figure 5). The measured areas of attachment are 20.9 mm^2^ above the macula, 9.7 mm^2^ over the optic nerve head, and 0.2 mm^2^ along the retinal vessel. All quantitative measurements are performed within the axial imaging range and transverse field of view of the long-wavelength SS-OCT instrument employed in this study.

**Figure 6:**
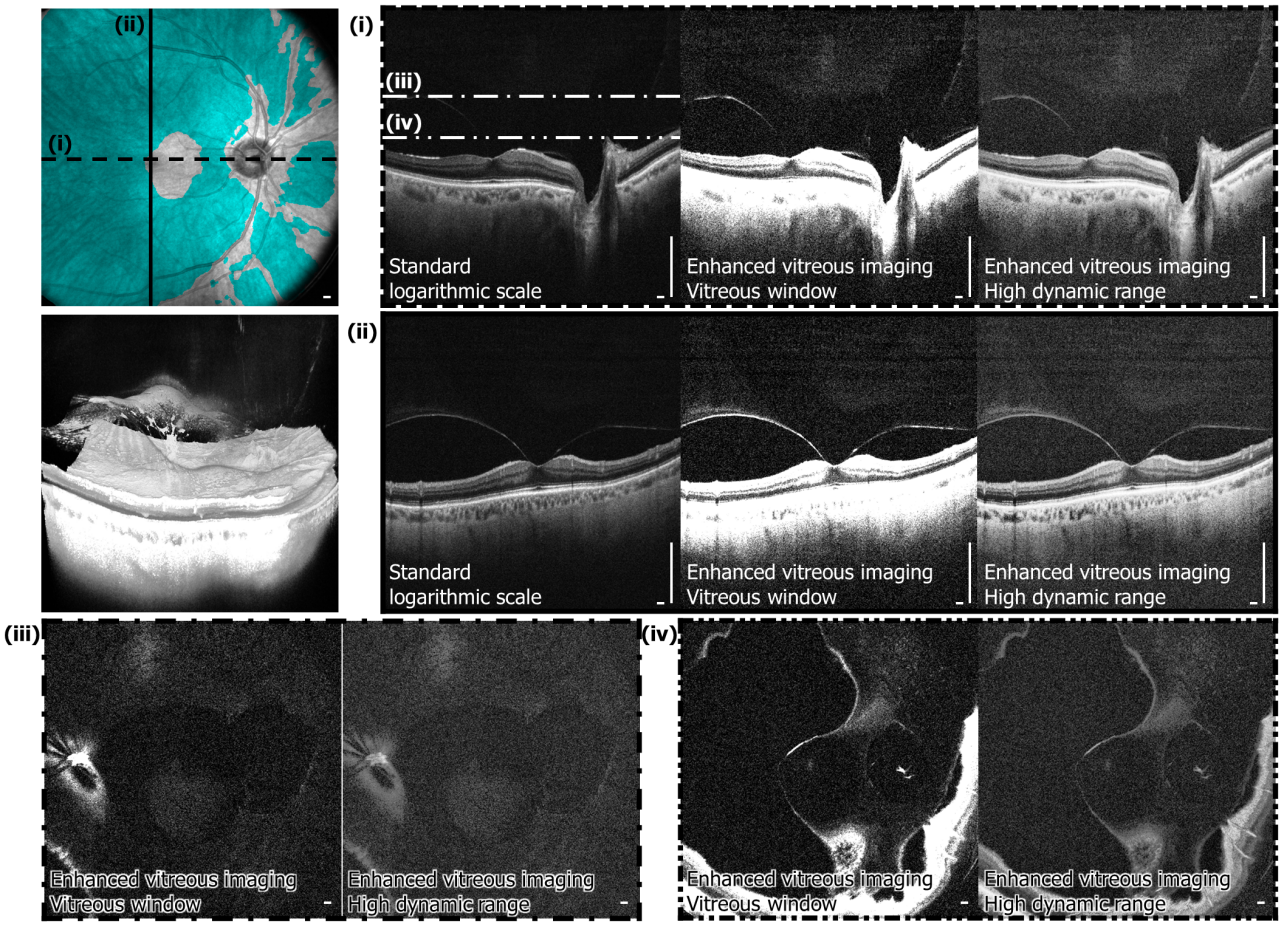
Enhanced vitreous imaging of vitreomacular traction (VMT). (Top left) Vitreous detachment map where the measured area of attachment is 4.4 mm^2^ over the macula and 23.2 mm^2^ over the optic nerve head and retinal vessels. (Second row left) Three-dimensional (3D) rendering of the long-wavelength, high-speed SS-OCT volumetric dataset. (i, ii, iii, iv) Selected cross-sectional images through the fovea and *en face* images are shown. Note the improved visualization of the contour and shape of the posterior hyaloid with enhanced vitreous imaging. The presence of vitreous fibers and hyperreflective reflective foci in the posterior vitreous are clearly visible in the *en face* images and three-dimensional rendering. Video S4 is a video animation of the rendering. Video S5 and S6 are cross-sectional and *en face* flythrough videos of the 3D volumetric dataset. Scale bars: 300 µm.

To demonstrate the potential utility of long-wavelength, high-speed volumetric SS-OCT enhanced vitreous imaging in vitreoretinal disease, a clinical example of vitreomacular traction (VMT) syndrome is displayed using the standard logarithmic scale versus the enhanced vitreous imaging methods (Figure 6). Images are from a 78-year-old male subject at the New England Eye Center who presented with visual distortion in his left eye. His best-corrected visual acuity was 20/40. Dilated fundus examination showed mild retinal pigment epithelium (RPE) changes, mild retinal thickening nasal to the fovea, and an epiretinal membrane (ERM). OCT imaging revealed that the patient had VMT syndrome: a partially-detached and thickened posterior hyaloid with persistent adhesion to the fovea, causing mild distortion of the foveal contour. Using the vitreous enhancement techniques applied to SS-OCT images, the contour and shape of the posterior hyaloid becomes clearly visible. *En face* images highlight the presence of individual vitreous fibers temporal to the macula; these fibers are also visualized in the 3D reconstruction of the volumetric dataset. The vitreous detachment map highlights that the vitreous remains attached at both the macula and optic nerve head, as well as along retinal arcade vessels and vessels nasal to the optic nerve. The measured areas of attachment are 4.4 mm^2^ over the macula and 23.2 mm^2^ over the optic nerve head and retinal vessels.

## Discussion

This study describes long-wavelength, high-speed SS-OCT enhanced vitreous imaging methods for improved OCT analysis of the posterior vitreous and vitreoretinal interface. Visualization of vitreous features is enhanced by processing standard logarithmic scale SS-OCT images using either a windowing technique, the “vitreous windowing display”, or a high-dynamic-range technique, “HDR display” for vitreous analysis and provides improved sensitivity for detection of vitreal and vitreoretinal features. Since processing is performed after the acquisition of standard logarithmic scale OCT images, these display methods could also be adapted to images from existing commercial SD-OCT instruments. However, SS-OCT has an advantage over SD-OCT for vitreous imaging because it maintains high sensitivity over a much longer imaging range and long wavelengths provide better immunity to ocular opacities. The high speed of SS-OCT combined with registration motion correction and merging enables wide-field volumetric imaging. As a result, long-wavelength, high-speed SS-OCT with enhanced vitreous imaging provides wide-field 3D information about the posterior vitreous structure, enabling detailed observations of the posterior vitreous and the vitreoretinal interface.

In this study, the non-overlapping 95% confidence intervals suggest a statistically significant improvement in overall detection sensitivity of posterior vitreous features using the vitreous windowing display as compared to the standard logarithmic scale display. While the HDR display improves the detection of some vitreal and vitreoretinal features when compared to the standard logarithmic scale display, the vitreous window display seems to provide improvement in detecting all of the features when compared to the standard logarithmic scale display and most of the features when compared to the HDR display. The HDR display is significantly worse in detecting granular opacities compared to the vitreous window display (30.7% vs 92.3%). This may be due to the blurring effect on very small features when applying the CLAHE filter, which is a known limitation of the HDR display process. Conversely, the HDR display was more sensitive in detecting hyaloid attachment to the retinal vessels (81.3% vs 75.0%) and the BPM (94.4% vs. 88.9%) when compared to the vitreous window display. The HDR display has the advantage that is preserves retinal contrast while enhancing visualization of the vitreous, which may be valuable in detecting interactions between the vitreous and the retina, such as at the attachment of the hyaloid to retinal vessels and fibrovascular vitreoretinal adhesions in diseases such as advanced diabetic retinopathy. In addition, the HDR display has the ability to visualize vitreous, retinal, and choroidal structures in a single image, similar to the combined depth imaging SD-OCT images shown by Barteselli *et al.* [21]

Prior studies have evaluated features in the posterior vitreous and vitreoretinal interface using commercial SD-OCT and SS-OCT instruments. Koizumi *et al.* presented 3D visualization of the VMT and idiopathic ERM using SD-OCT over a 6 mm ×6 mm retinal area where hyperreflectivity of the detached posterior hyaloid was observed. [5] Mojana *et al.* demonstrated the ability of SD-OCT to image the physiologic and pathologic vitreous structure to provide detailed analysis of the vitreoretinal interface. [22] The authors found that SD-OCT showed a higher prevalence of PVD compared to clinical slit-lamp biomicroscopy examination as well as improved visualization of vitreal details such as the BPM, area of Martegiani, Bergmeister papilla, hyaloid adhesion to retinal vessels, hyperreflective vitreous strands, and granular opacities (described as insertion of anteroposterior fibers or cellular aggregations). Using single 9 mm nasal-temporal and 6 mm superior-inferior cross-sectional SD-OCT scans in normal eyes, Shimada *et al.* described the size and dimension of the BPM and its separation from the pre-papillary Cloquet's canal by a thin wall. [23] Itakura and Kishi further confirmed the presence of BPM in all ages and observed thickened vitreous cortex and perifoveal posterior vitreous detachment in older individuals by adjusting the contrast in single 9 mm nasal-temporal SD-OCT cross-sectional images, similar to the “windowing” approach performed in the present study. [14] Recent results from Itakura *et al.* showed clarified boat-shaped BPM structure *in vivo* using single 12 mm nasal-temporal and 12 mm superior-inferior SS-OCT scans where brightness and contrast of the images were adjusted to enhance the vitreous. [11] In addition, Stanga *et al.* performed anatomical characterization of the cortical vitreous and vitreoretinal interface using the single 12 mm nasal-temporal SS-OCT scans by measuring the width and depth of the BPM and classifying the degree of posterior vitreous detachment. [11], [12]

Nevertheless, the analyses in previous studies were either restricted to isolated cross sectional images or 3D datasets with inadequate cross-sectional image quality as well as limited retinal coverage. The present study is to our knowledge, the first to investigate volumetric imaging with high A-scan density and demonstrate that posterior vitreous structure and the vitreoretinal interface can be visualized in 3D using long-wavelength, high-speed, wide-field motion-corrected volumetric SS-OCT datasets and enhanced vitreous imaging. Enhanced vitreous imaging using SS-OCT enables detailed examination of vitreal and vitreoretinal features over a large volume of the posterior vitreous. In addition, 3D datasets allow detailed analysis of physiological vitreous spaces such as the BPM and the Cloquet's canal. Moreover, mapping of the locus and area of vitreous detachment from the retina and measurement of the area of vitreoretinal adhesion can also be accomplished in normal eyes as well as eyes with vitreoretinal disease, as demonstrated in an eye with vitreomacular traction syndrome. Such measurements and maps may be helpful to follow vitreous detachment over time, for preoperative planning, and/or to assess treatment response to surgery or enzymatic vitreolysis.

A limitation of this study is that only subjects with healthy eyes were included. Studies in diseased eyes are currently underway and will help better understand the clinical significance of features observed in the posterior vitreous and vitreoretinal interface. Another limitation of this study is the imaging speed and imaging range of our prototype SS-OCT instrument. Our current prototype SS-OCT already achieves ∼4× higher imaging speed (100,000 A-scans/second), ∼80% longer imaging range (3.6 mm), and ∼2× transverse field of view (12 mm×12 mm) than commercial SD-OCT instruments. However, since the vitreous is a dynamic tissue and its shape and configuration may be altered with eye movement and position, it is possible that the vitreous anatomy changes during the time interval between acquisitions of multiple 3D-OCT datasets [15], which may result in blurring of certain vitreal and vitreoretinal features when the registration motion-correction algorithm is applied. Furthermore, although the current instrument has a long imaging range and wide field of view, visualization of structures such as the BPM, area of Martegiani, and vitreous attachment to the retinal vessels in our study is limited by the axial imaging range and transverse field of view of the prototype SS-OCT instrument. Improved field of view could be achieved by creating montage OCT images as described by Mori *et al*, however this would increase imaging time and would limit the ability to create a detailed 3D dataset. [24] Future SS-OCT instruments with higher speeds, faster image acquisition, improved sampling, and longer imaging range will be able to help visualize more of the vitreal and vitreoretinal features in both the transverse and anteroposterior dimensions. [25] A final limitation of the study was that 3D processing of images was performed manually, which was time consuming. Future development of computer image analysis algorithms will enable rapid, real time mapping of vitreous detachment.

In conclusion, this prospective cross-sectional study demonstrates improved visualization of details of the posterior vitreous and the vitreoretinal interface in normal eyes using long-wavelength, high-speed, SS-OCT enhanced vitreous imaging. SS-OCT enhanced vitreous imaging may be useful in applications that require detailed analysis of microstructural details of the vitreous in healthy and diseased eyes. SS-OCT enhanced vitreous imaging may be particularly promising as a new tool for imaging the 3D structure of the vitreous in patients with disorders of the vitreomacular interface and assessing treatment response after vitrectomy or pharmacologic vitreolysis.

## Supporting Information

Video S1 **Cross-sectional flythrough video of the 3D volumetric dataset shown in** **Figure 2** **.**
(MOV)

Video S2 ***En face*** **flythrough video of the 3D volumetric dataset shown in** **Figure 2** **.**
(MOV)

Video S3 **3D rendering video animation of the dataset shown in** **Figure 4** **, illustrating the retina along with the** ***bursa premacularis*** **(BPM) and area of Martegiani highlighted above.**
(MOV)

Video S4 **3D rendering Video animation of the dataset shown in** **Figure 6** **.**
(MOV)

Video S5 **Cross-sectional flythrough video of the 3D volumetric dataset shown in** **Figure 6** **.**
(MOV)

Video S6 ***En face*** **flythrough video of the 3D volumetric dataset shown in** **Figure 6** **.**
(MOV)
